# Association Between Diabetes and Increased Prevalence of Paranasal Sinus Disease: A Cross-Sectional Study in Japanese Adults

**DOI:** 10.2188/jea.JE20140163

**Published:** 2015-04-05

**Authors:** Yusuke Kabeya, Kiyoe Kato, Masuomi Tomita, Takeshi Katsuki, Yoichi Oikawa, Akira Shimada

**Affiliations:** Department of Internal Medicine, Tokyo Saiseikai Central Hospital, Tokyo, Japan; 東京都済生会中央病院 内科

**Keywords:** diabetes, glycemic status, paranasal sinus disease

## Abstract

**Background:**

The association between diabetes and paranasal sinus disease has not been thoroughly investigated.

**Methods:**

We cross-sectionally investigated the association between diabetes and the presence of paranasal sinus disease, which was confirmed by a head MRI scan in 1350 adults who underwent a health screening program focusing on brain diseases and metabolic syndrome. Logistic regression, which was adjusted for age, sex, body mass index, waist-to-hip ratio, hypertension, smoking status, alcohol intake, and white blood cell count, was performed to calculate the odds ratio (OR) of having paranasal sinus disease among adults with diabetes in relation to those without. The dose-response relationship between hemoglobin A1c (HbA1c) levels and the presence of paranasal sinus disease was also investigated.

**Results:**

Of the 1350 participants (mean age, 61.6 ± 10.0 years; 71.6% men), 220 diabetes cases were identified. Paranasal sinus disease was diagnosed in 151 adults. The adjusted OR of having paranasal sinus disease was 1.74 (95% confidence interval [CI], 1.12–2.71) in those with diabetes. The odds of having paranasal sinus disease increased with HbA1c levels. Compared to those with HbA1c of ≤5.4%, those with HbA1c of 5.5%–6.4%, 6.5%–7.9%, and ≥8.0% were more likely to have paranasal sinus disease, with adjusted ORs of 1.32 (95% CI, 0.88–1.98), 1.63 (95% CI, 0.86–3.09) and 2.71 (95% CI, 1.12–6.61), respectively (*P* for trend = 0.019).

**Conclusions:**

Diabetes may be significantly associated with higher prevalence of paranasal sinus disease in Japanese adults. We should keep this increased risk in mind when a diabetic patient is suspected of having paranasal sinus disease.

## Abstract (ja)

**【背景】:**

糖尿病と副鼻腔病変の関連は過去の報告は少ない。この研究では日本人成人の健診集団において糖尿病の有無・血糖値の状態と副鼻腔病変の有病率の関連を横断的に調査した。

**【方法】:**

東京都済生会中央病院において2007年から2011年に健康診断を受け、かつその際に頭部MRI検査を施行している40歳以上の成人1350例を対象とした。糖尿病は、質問票による糖尿病の自己申告と採血データによって診断し、副鼻腔病変は頭部MRIにて副鼻腔に粘膜肥厚、ポリープ、液体貯留があるものを副鼻腔病変の有所見者とした。ロジスティック回帰分析を用いて、糖尿病があることによる副鼻腔病変のオッズ比を算出した。また、HbA1cの値と副鼻腔病変のオッズ比も算出し血糖値の状態との用量依存性を検証した。これらのオッズ比は年齢、性別、BMI、ウェスト・ヒップ比、高血圧、喫煙、飲酒、白血球数にて調整を行った。

**【結果】:**

1350名（平均61歳、男性72％）のうち220名が糖尿病を有しており、副鼻腔病変を有する者は151名であった。糖尿病があることによる副鼻腔病変を有する調整後オッズ比は1.74（95%信頼区間　1.27-2.71）であった。また、HbA1cが5.5%未満の人を基準として5.5-6.4%、6.5-7.9%、8%以上の人では、副鼻腔病変を有する調整後オッズ比は、各1.32（95%信頼区間　0.88-1.98）、1.63（95%信頼区間　0.86-3.09）、2.71（95%信頼区間　1.12-6.61）であった（傾向検定 P=0.019）。

**【結論】:**

この研究にて、日本人成人において糖尿病を有することと副鼻腔病変の有病率が高いことの関連が示唆された。また、血糖値の状態と副鼻腔病変の有病率に正の用量依存関係を認めた。副鼻腔疾患を疑う場合、糖尿病者・血糖値が高い症例ではその有病率が高いことを念頭に診療にあたるべきであろう。

## INTRODUCTION

Paranasal sinus disease develops as a result of inflammation of the sinus cavities. Due to the widespread use of a head magnetic resonance imaging (MRI) or computed tomography (CT) scan, which routinely include the paranasal sinuses, incidental abnormalities of the paranasal sinuses are frequently detected. Although the clinical significance of the incidental findings remains controversial, an appropriate work-up was proposed for patients with suspected paranasal sinus disease.^1^ There have been several studies^1^^–^^6^ that reported the prevalence of paranasal sinus disease in a variety of asymptomatic populations. A large variation was observed in the prevalence of paranasal sinus disease on radiological examinations, ranging from approximately 13% to 63%. Clinical characteristics related to the incidental findings have been also investigated.^3^^,^^7^^,^^8^ However, they have not been fully elucidated.

Patients with diabetes are considered susceptible to infections.^9^ Although a causal relationship has not been confirmed, there is clinical evidence of an association between diabetes and respiratory tract infections. One survey^10^ demonstrated that the number of general practitioner consultations for respiratory tract infections was higher in patients with diabetes than those without. Hyperglycemia alters immune function,^11^ which makes a host susceptible to infections. Changes in the microcirculation due to chronic hyperglycemia impair the supply of nutrition and oxygen to tissues,^12^ which might affect the ability to control infections. Ciliary motility in respiratory epithelium, which is a key defense function for protecting a host against invading pathogens, is also important.^12^ Considering these mechanisms, those with diabetes are expected to be more likely to have paranasal sinus disease than those without. However, few studies have investigated the association.

In the present study, we hypothesized that diabetes is associated with increased prevalence of paranasal sinus disease and tested the hypothesis in a Japanese adult population.

## METHODS

### Study participants

This was a cross-sectional study performed at the medical checkup unit of Saiseikai Central Hospital in Tokyo, Japan. Several studies have been already performed with the same protocol and were reported elsewhere.^13^^,^^14^ In brief, the study population was composed of participants aged 40 years or older (*n* = 1351) who underwent a health screening program focusing on brain diseases and metabolic syndrome from January 2007 to December 2011. They were generally residents of neighboring areas and visited the hospital not for symptomatic diseases but for a health check. Of the 1351 participants, 1 participant was excluded because of missing data. The study protocol was reviewed and approved by the ethics committee of Saiseikai Central Hospital.

### Anthropometric and laboratory measurements

Information on age, sex, weight, height, body mass index (BMI), waist to hip ratio, blood pressure, white blood cell (WBC) count, plasma glucose levels, and hemoglobin A1c (HbA1c) levels was gathered from the health screening data. Blood pressure was measured in a sitting posture using an automated sphygmomanometer (Udex-Twin; ELK Corp, Osaka, Japan). Blood samples and anthropometric measurements were obtained 12 hours or more after the last caloric intake. Plasma glucose levels were measured using a GA08 automated glucose analyzer (A&T Co., Kanagawa, Japan). HbA1c levels were measured using an HPLC analyzer (HLC-723G8; Tosoh, Tokyo, Japan). The HbA1c data were converted to the equivalent values of the National Glycohemoglobin Standardization Program according to the statement of the Japan Diabetes Society.^15^ Information on smoking status, alcohol intake, use of anti-diabetic medications, and use of anti-hypertensive medications was obtained by a self-administered questionnaire. Hypertension was defined as any of the following: use of anti-hypertensive medications based on the questionnaire, systolic blood pressure ≥140 mm Hg, or diastolic blood pressure ≥90 mm Hg. The participants were categorized into three groups (non-smoker, past smoker, or current smoker) according to their smoking status. Regarding alcohol intake, the participants were categorized into three groups according to their frequency of alcohol intake (abstainer, 1–4 days per week, or 5 days or more per week).

### Definitions of diabetes

Diabetes was defined as any of the following: self-reported diabetes or use of anti-diabetic medications based on the questionnaire, fasting plasma glucose (FPG) ≥126 mg/dL, or HbA1c ≥6.5%.

### Paranasal sinus disease

A head MRI scan was performed using a 1.5-tesla scanner (SIGNA HDxt 1.5T; GE Healthcare Japan, Tokyo, Japan). The images were analyzed by a trained neurologist who did not know the purpose of the present study. Paranasal sinus disease was defined as the presence of abnormal findings (mucosal thickening, polyps, or sinus opacification) in at least one of the paranasal sinuses detected by a head MRI scan.

### Statistical analysis

Characteristics of the participants were compared between those with paranasal sinus disease and those without. Student’s unpaired *t* tests were used for comparing continuous variables, while χ^2^ tests were used for comparisons between categorical variables. Regarding WBC count, the log-transformed values were compared to normalize distribution. The prevalence of paranasal sinus disease was calculated according to the presence of diabetes and was standardized to the 1985 model population in Japan.

Then, the association between HbA1c and the prevalence of paranasal sinus disease was graphically and statistically examined using a restricted cubic spline model, where observed and expected prevalence in each HbA1c level was plotted (Figure). The model confirmed that the log-odds of having paranasal sinus disease were a linear function of HbA1c levels.

**Figure.**
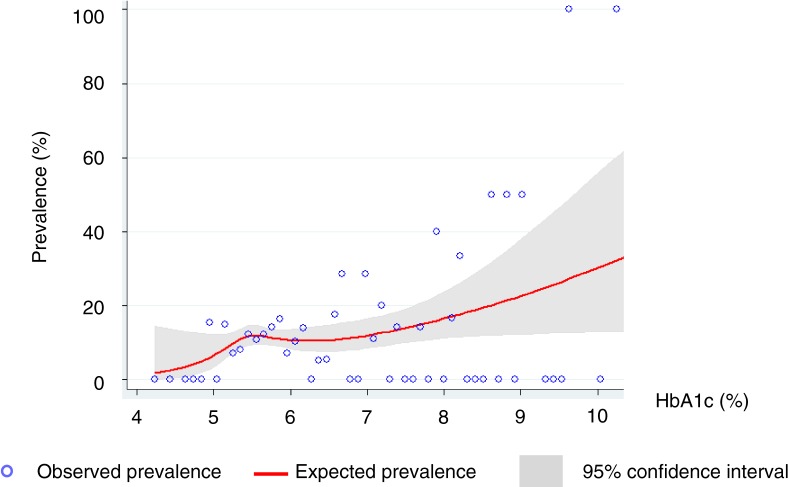
The prevalence of paranasal sinus disease by HbA1c levels.

Finally, multiple logistic regression analysis was performed to calculate the odds ratios (ORs) and the 95% confidence intervals (CIs) of having paranasal sinus disease in those with diabetes in relation to those without. The ORs and the 95% CIs were adjusted for age, sex, BMI, waist-to-hip ratio, hypertension, smoking status, alcohol intake, and WBC count. In addition, the dose-response relationship, which was adjusted for the same variables, was examined between HbA1c levels and the prevalence of paranasal sinus disease. HbA1c levels were analyzed as a categorical variable (HbA1c of ≤5.4%, 5.5%–6.4%, 6.5%–7.9% and ≥8.0%) and a continuous variable. Statistical analyses were performed using STATA software version 11 (StataCorp, College Station, TX, USA). All statistical tests were two-sided, and *P*-values less than 0.05 were considered statistically significant.

## RESULTS

Of the 1350 participants (mean age, 61.6 ± 10.0 years; 71.6% men), 220 diabetes cases were identified. Paranasal sinus disease was diagnosed in 151 adults. Table 1 shows the characteristics of study participants who had paranasal sinus disease and those who did not. Those with paranasal sinus disease were younger and more likely to be male than those without. They had higher levels of FPG and HbA1c, resulting in higher prevalence of diabetes (23.2% vs 15.4%). Systolic blood pressure was not significantly different between the groups, while diastolic blood pressure was higher in those with paranasal sinus disease. However, when the information on use of anti-hypertensive medications was taken into account, the proportions of those who had hypertension were not significantly different between the groups. The habits of smoking and alcohol intake were more common in those with paranasal sinus disease than in those without. WBC count and waist-to-hip ratios appeared higher in those with paranasal sinus disease; however, the differences were moderate and showed borderline significance.

**Table 1.** Characteristics of study participants

	Total (*n* = 1350)	Paranasal sinus disease		*P* for difference

		No (*n* = 1199)	Yes (*n* = 151)	
Age, years	61.6 (10.0)	61.8 (10.1)	59.9 (9.1)	0.024
Males, *n* (%)	966 (71.6)	842 (70.2)	124 (82.1)	0.002
BMI, kg/m^2^	23.4 (3.2)	23.3 (3.2)	24.1 (3.1)	0.001
Waist to hip ratio	0.90 (0.07)	0.90 (0.07)	0.91 (0.07)	0.058
Systolic blood pressure, mm Hg	122 (19)	123 (19)	124 (19)	0.570
Diastolic blood pressure, mm Hg	77 (12)	76 (12)	78 (12)	0.034
Fasting plasma glucose, mg/dL	107 (23)	106 (22)	110 (27)	0.034
HbA1c, %	5.8 (0.8)	5.8 (0.7)	6.0 (0.9)	0.025
WBC^a^, count/µL	5400 (5320–5470)	5370 (5290–5450)	5590 (5340–5840)	0.089

Smoking status, *n* (%)				
non-smoker	764 (56.6)	690 (57.6)	74 (49.0)	0.034
past smoker	417 (30.9)	368 (30.7)	49 (32.5)	
current smoker	169 (12.5)	141 (11.8)	28 (18.5)	
Alcohol intake, *n* (%)				
abstainer	273 (20.2)	249 (20.8)	24 (15.89)	0.008
1–4 days per week	620 (45.9)	561 (46.8)	59 (39.07)	
5 days or more per week	457 (33.9)	389 (32.4)	68 (45.0)	
Hypertension, *n* (%)	531 (39.3)	469 (39.1)	62 (41.1)	0.645
Diabetes, *n* (%)	220 (16.3)	185 (15.4)	35 (23.2)	0.015

BMI, body mass index; HbA1c, hemoglobin A1c; WBC, white blood cell.

Data are presented as mean (standard deviation) for continuous variables and count (%) for categorical variables except WBC.

^a^WBC count is presented as geometric mean (95% confidence interval) because of its skewed distribution.

Table 2 shows age-specific prevalence of paranasal sinus disease according to the presence of diabetes. In general, the prevalence of paranasal sinus disease was higher among those with diabetes than those without in each age category. The prevalence appeared to decrease with age. The age-standardized prevalence of paranasal sinus disease was 11.9% in the overall population of the study; prevalence was 18.8% in adults with diabetes and 11.0% in those without.

**Table 2.** Prevalence of paranasal sinus disease according to the presence of diabetes

Age category, years	Total (*n* = 1350)			Diabetes					

				No (*n* = 1130)			Yes (*n* = 220)		
		
	paranasal sinus disease	Number of subjects	prevalence (%)	paranasal sinus disease	Number of subjects	prevalence (%)	paranasal sinus disease	Number of subjects	prevalence (%)
≤49	26	192	13.5	25	187	13.4	1	5	20.0
50–54	21	158	13.3	17	144	11.8	4	14	28.6
55–59	28	222	12.6	23	185	12.4	5	37	13.5
60–64	34	269	12.6	27	216	12.5	7	53	13.2
65–69	19	243	7.8	12	195	6.2	7	48	14.6
70≤	31	396	7.8	12	203	5.9	11	63	17.5

Crude prevalence	151	1350	11.2	116	1130	10.3	35	220	15.9
Age-standardized prevalence^a^			11.9			11.0			18.8

^a^Standardized to the 1985 Japanese model population.

Regarding glycemic measures, the association between HbA1c levels and the prevalence of paranasal sinus disease was demonstrated (Figure), where the prevalence of paranasal sinus disease increased with HbA1c levels.

In the logistic regression analysis (Table 3), a significant association was observed between diabetes and the presence of paranasal sinus disease after adjustment for multiple variables (OR 1.74; 95% CI, 1.12–2.71). The association between the presence of paranasal sinus disease and HbA1c levels was also observed. In relation to those with HbA1c of ≤5.4%, those with HbA1c of 5.5%–6.4%, 6.5%–7.9%, and ≥8.0% were more likely to have paranasal sinus disease, with ORs of 1.32 (95% CI, 0.88–1.98), 1.63 (95% CI, 0.86–3.09), and 2.71 (95% CI, 1.12–6.61), respectively (*P* for trend = 0.019). When HbA1c was analyzed as a continuous variable, a 1% absolute increase in HbA1c levels was associated with 1.28-fold increased odds of having paranasal sinus disease.

**Table 3.** The odds ratios of having paranasal sinus disease according to the presence of diabetes or HbA1c levels

	Crude OR	95% CI	Adjusted OR^a^	95% CI
Diabetes				
No	1.00	reference	1.00	reference
Yes	1.65	1.10–2.49	1.74	1.12–2.71

HbA1c levels (Categorical)				
≤5.4%	1.00	reference	1.00	reference
5.5–6.4%	1.24	0.85–1.82	1.32	0.88–1.98
6.5–7.9%	1.51	0.83–2.74	1.63	0.86–3.09
≥8.0%	2.47	1.07–5.70	2.71	1.12–6.61
	(*P* for trend = 0.026)		(*P* for trend = 0.019)	
(Continuous)				
1% absolute increase	1.25	1.03–1.52	1.28	1.03–1.59

CI, confidence interval; HbA1c, hemoglobin A1c; OR, odds ratio.

^a^The model was adjusted for age, sex, body mass index, waist to hip ratio, hypertension, white blood cell count, smoking status, and alcohol intake.

## DISCUSSION

The present study examined the association between diabetes and paranasal sinus disease. We found that the prevalence of paranasal sinus disease was higher in those with diabetes than those without. The logistic regression analysis revealed that participants with diabetes had 1.74-fold increased odds of having paranasal sinus disease compared to those without. The odds of having paranasal sinus disease also increased with HbA1c levels. The dose-response relationship further strengthened the positive association between glycemic status and the presence of paranasal sinus disease.

Paranasal sinus disease may be caused by multiple factors, including infections, allergic reactions, exposure to irritant substances such as smoking, and impairment of mucus clearance. Although direct evidence of the association between diabetes and paranasal sinus disease is scarce, the present study suggests that diabetes and its related conditions could affect these factors and accelerate the development of paranasal sinus disease.

As for infections, it is well known that adults with poorly controlled diabetes are susceptible to a variety of infections.^9^ Normal activities of macrophages and neutrophils, such as chemotaxis, adherence, phagocytosis, and intracellular killings of microbes, are impaired under hyperglycemic conditions.^16^ Evidence on the association between diabetes and respiratory tract infections has also been accumulating. Increased frequency and severity of respiratory tract infections among those with diabetes has been reported. One prospective cohort study^17^ reported that patients with type 2 diabetes had a 1.3-fold higher risk of lower respiratory tract infections compared to non-diabetic patients. Higher risk of bacteremic pneumococcal pneumonia was also observed in diabetic patients.^18^ A meta-analysis^19^ that analyzed 33 000 patients with community acquired pneumonia showed that patients with diabetes had higher mortality than those without diabetes. Although the paranasal sinuses are a part of the respiratory system, few studies have assessed the risk of paranasal sinus infections among adults with diabetes. When considering the mechanisms of susceptibility of adults with diabetes to respiratory tract infections, it might be reasonable to apply the evidence on the association between diabetes and respiratory tract infections to the association of diabetes with paranasal sinus infections.

Ciliary motility is also important for normal clearance of mucus and invading pathogens from the sinus cavities.^12^ Although evidence is scarce, changes in respiratory tract epithelial cells have been observed in human and animal studies of diabetes.^20^^–^^22^ Such changes could affect the functions of ciliary motility and impair mucus clearance, resulting in the development of paranasal sinus diseases.

Regarding allergic reactions, one previous study^23^ demonstrated that hyperglycemia is associated with lower prevalence of allergic rhinitis. From this finding, it is expected that paranasal sinus disease due to allergic reactions could be less common in adults with diabetes, which is contrary to the findings in the present study. The reason for the conflicting results remains unclear. There is a possibility that the contribution of allergic reactions to the development of paranasal sinus diseases could be minimal compared with that of infections or other factors. In addition, it remains unclear whether adults with symptoms of allergic rhinitis are more likely to have sinus findings on a head MRI. Further research is required to elucidate the mechanisms.

Although the prevalence of paranasal sinus disease detected by a head MRI scan was higher among those with diabetes than those without, the clinical importance of the increased prevalence remains unclear. Some studies^6^^,^^24^ have reported that incidental sinus abnormalities on radiological examinations do not have clinical significance. However, it is possible that some asymptomatic cases could lead to serious diseases or eventually progress to clinically symptomatic illness. Given the scarcity of evidence, it might be reasonable to pay attention to clinical symptoms when a patient is suspected of having paranasal sinus disease. Moreover, if a patient has diabetes, the increased risk of paranasal sinus disease should be taken into consideration when making a diagnosis.

Several limitations in the present study should be mentioned. First, the participants were selected from a cohort of patients who underwent a health screening program focusing on brain diseases and metabolic syndrome. These participants may be more health-conscious than the general population, which could limit the generalizability of the study. Second, it has been reported that the volume of normal sinus mucosa changes periodically, indicating that some of the abnormalities detected by a head MRI scan may not be abnormal findings but physiological changes. This misclassification is considered to occur randomly, which could dilute the risk estimates of the association between diabetes and paranasal sinus disease. Third, we cannot confirm the causal relationship, since the study design was cross-sectional. In addition, there could be unmeasured confounders, such as history of allergic rhinitis or socioeconomic status. As for the direction of the causal relationship, no study has reported that the presence of paranasal sinus diseases could contribute to the development of diabetes. On the other hand, there has been plausible evidence that diabetes may contribute to the development of paranasal sinus diseases, which we mentioned above. Further research is required to confirm the causal relationship. Despite these limitations, this is the first study to report an association between diabetes and paranasal sinus disease. The multivariate analysis enabled us to adjust the association for possible confounders. The definition of diabetes was confirmed by a questionnaire and laboratory data, and paranasal sinus disease was diagnosed with a head MRI scan. The use of accurate methods for measuring these variables minimized measurement errors, which might have contributed to the significant association in the present study.

In conclusion, the present study found that adults with diabetes had increased prevalence of paranasal sinus disease. A dose-response relationship between hyperglycemia and the presence of paranasal sinus disease was also observed. Although the clinical significance of the findings detected by a head MRI scan remains undetermined, we should keep the increased prevalence in mind when a diabetic patient is suspected of having paranasal sinus disease in daily medical practice.

## ONLINE ONLY MATERIAL

Abstract in Japanese.
