# Supplementary material for: Association Between Diabetes and Increased Prevalence of Paranasal Sinus Disease: A Cross-Sectional Study in Japanese Adults
Source: J Epidemiol. 2015 Apr 5;25(4):297–302. doi: 10.2188/jea.JE20140163 (PMC4375284; doi:10.2188/jea.JE20140163)
Supplement: Abstract in Japanese. [file je-25-297-s001.pdf]

糖尿病と副鼻腔病変の有病率の関連：日本人成人健診集団における横断研究

壁谷悠介 1、加藤清恵 1、富田益臣 1、香月健志 1、及川洋一 1、島田朗 1

1 東京都済生会中央病院　内科

【背景】糖尿病と副鼻腔病変の関連は過去の報告は少ない。この研究では日本人成人の健診集団において糖尿病の有無・血糖値の状態と副鼻腔病変の有病率の関連を横断的に調査した。

【方法】東京都済生会中央病院において 2007 年から 2011 年に健康診断を受け、かつその際に頭部 MRI 検査を施行している 40 歳以上の成人 1350 例を対象とした。糖尿病は、質問票による糖尿病の自己申告と採血データによって診断し、副鼻腔病変は頭部 MRI にて副鼻腔に粘膜肥厚、ポリープ、液体貯留があるものを副鼻腔病変の有所見者とした。ロジスティック回帰分析を用いて、糖尿病があることによる副鼻腔病変のオッズ比を算出した。また、HbA1c の値と副鼻腔病変のオッズ比も算出し血糖値の状態との用量依存性を検証した。これらのオッズ比は年齢、性別、BMI、ウェスト・ヒップ比、高血圧、喫煙、飲酒、白血球数にて調整を行った。

【結果】1350 名（平均 61 歳、男性 72%）のうち 220 名が糖尿病を有しており、副鼻腔病変を有する者は 151 名であった。糖尿病があることによる副鼻腔病変を有する調整後オッズ比は 1.74（95%信頼区間　1.27-2.71）であった。また、HbA1c が 5.5%未満の人を基準として 5.5-6.4%、6.5-7.9%、8%以上の人では、副鼻腔病変を有する調整後オッズ比は、各 1.32（95%信頼区間　0.88-1.98）、1.63（95%信頼区間　0.86-3.09）、2.71（95%信頼区間　1.12-6.61）であった（傾向検定 P=0.019）。

【結論】この研究にて、日本人成人において糖尿病を有することと副鼻腔病変の有病率が高いことの関連が示唆された。また、血糖値の状態と副鼻腔病変の有病率に正の用量依存関係を認めた。副鼻腔疾患を疑う場合、糖尿病者・血糖値が高い症例ではその有病率が高いことを念頭に診療にあたるべきであろう。

キーワード：糖尿病、血糖値の状態、副鼻腔病変
